# 

**Published:** 1940

**Authors:** 


### CONTENTS.

| | Page |
|---|---|
| **Cerebro-Spinal Meningitis in Bristol.** By B. A. Peters, M.D., D.P.H. | 77 |
| **Traumatic Extradural Hæmorrhage. Part I.** By A. Rendle Short, M.D., B.S., B.Sc., F.R.C.S., and Marjorie Dunster, M.B., Ch.B. | 81 |
| **Traumatic Extradural Hæmorrhage. Part II.** *The Clinical Application of Electro-Encephalograpy.* By W. Grey Walter, M.A. | 86 |
| **Head Injuries in Wartime.** By F. Wilfred Willway, M.D., M.S., F.R.C.S. | 91 |
| **Reviews of Books** | 99 |
| **Editorial Note** | 101 |
| **The Medical Library of the University of Bristol** | 102 |
| **Publications Received** | 104 |
| **Local Medical Notes** | 105 |

